# Vertex-Oriented Method for Polyhedral Reconstruction of 3D Buildings Using OpenStreetMap

**DOI:** 10.3390/s24247992

**Published:** 2024-12-14

**Authors:** Hanli Liu, Carlos J. Hellín, Abdelhamid Tayebi, Francisco Calles, Josefa Gómez

**Affiliations:** Department of Computer Science, Universidad de Alcalá, 28805 Alcalá de Henares, Spain; hanli.liu@edu.uah.es (H.L.);

**Keywords:** 3D city modeling, OpenStreetMap, 3D reconstruction

## Abstract

This work presents the mathematical definition and programming considerations of an efficient geometric algorithm used to add roofs to polyhedral 3D building models obtained from OpenStreetMap. The algorithm covers numerous roof shapes, including some well-defined shapes that lack an explicit reconstruction theory. These shapes include gabled, hipped, pyramidal, skillion, half-hipped, gambrel, and mansard. The input data for the developed code consist of latitude and longitude coordinates defining the target area. Geospatial data necessary for the algorithm are obtained through a request to the overpass-turbo service. The findings showcase outstanding performance for buildings with straightforward footprints, but they have limitations for the ones with intricate footprints. In future work, further refinement is necessary to solve the mentioned limitation.

## 1. Introduction

The research on 3D reconstruction has always been a challenging goal, serving as a scientific problem and core technology across various fields. Among these, urban area reconstruction involves a broad spectrum of techniques, including point mining and utilizing diverse information sources such as aerial imagery [1] and street view [2]. This study reconstructs urban areas using information gathered from OpenStreetMap’s (OSM) data mining tool [3]. The authors recently introduced an innovative method for reconstructing 3D building maps [4]. Specifically, this study concentrates on analyzing the shapes of building roofs.

The techniques of 3D model generation are frequently used in a wide range of fields, being applied by mixing different tools and information sources, allowing a good visualization of the environmental elements.

Focusing on the approach related to this paper, in the case of urban areas, the paramount element is the vertex positioning of the buildings, which is the basis for the 3D model visualization. Furthermore, modeling of other aspects like ground elevation, area water flow, representative vegetation, and building face forms can be added to generate the models. All of those details can be scaled by the Level of Detail (LoD) required in the model application [5,6], adapting to the computational and economic aspects of the user. The 3D models with only vertex and face composition have the lowest LoD (LoD 1).

Numerous studies use data from OSM. In the work of Girindran et al., multiple open data including OSM are mixed for the foundation of the 3D models [7]. Alomía et al. made a case study on integrating a procedurally generated road network with the geographic information from OSM into the same model [8]. The tool of Egea-Lopez et al. generates interactive 3D environments, using data directly from OSM and relying on its integration with the Unity game engine [9].

Typically, direct manipulation of vertices is unnecessary. Mesh definitions often provide a means to streamline the computational processes, where specified parameters of known defined volumes (such as cylinders, spheres, etc.) can compose these vertices. Using these definitions does not impede the incorporation of optimizations, as outlined in [10].

To create 3D urban models, the initial prerequisite is the availability of one or more sources of urban information for processing. Various possibilities exist, and a discussion on some of these sources will follow promptly.

In today’s digital landscape, the swiftest means of accessing information online involves harnessing the vast data repositories offered by existing data mining tools, such as Overpass [3], utilized in this study. These tools streamline data acquisition in diverse formats, including commonly used ones like GeoJSON and GPX. Some standardized data models are generally used to store information, such as CityGML [6].

Another commonly utilized technique involves gathering information from images, including directly capturing photographs from street views [2]. While the information obtained may pose challenges for human comprehension, it proves valuable for deep learning techniques.

In a more precise way, specific attributes like the roof shapes examined in this study can be identified. Techniques such as classification using orthophotos [11], commonly stored in datasets for subsequent analysis, enable the differentiation of various roof forms.

Complex processes involve not only extracting the coordinates of the building’s exterior but also employing automated methods to directly extract roof surfaces. This is achieved through the integration of Light Detection and Ranging (LiDAR) data and multispectral imagery [12]. By detecting and extracting roof lines from images, forming faces, and estimating their inclinations, these methods streamline the process.

Overall, owing to the diverse specifications of each technique, it is feasible to employ a combination of methods or sources to complement one another, for example, fusing 3D mesh data from unmanned aerial vehicles with a LiDAR point cloud [13] and integrating a building information model with a real-scene 3D model [14].

The 3D models obtained are fundamental for different designs with their specific purposes. One of the uses of the results of this work is to simulate radio propagation in 3D space [15,16]. The smart cities need the 3D models for significant applications like surveying and mapping [10]. The creation of a digital twin for digital analysis or research on the buildings needs to be based on 3D models, to be viewable at all levels, for example, for the preservation of historic buildings [17] and structure health monitoring [14].

Presently, within the official 3D web viewer of OSM [18], non-flat roofs are predominantly interpreted as having a gabled shape. This refers to the simplest form with only two inclined roof faces, characterized as a “roof with ridge”. The managed shapes are cone, dome, pyramid, skillion, round, and onion. The 3D model faces are obtained by direct composition of triangles. All the model components of polygon, quadrilateral, cylinder, circle, dome, and pyramid have their functions to create the triangle composition.

The work of Župan et al. [19] is an example of mixing LiDAR data with OSM, which applies fills to required missing attributes, focused on roof heights and basic shapes distinguished (between flat, pitched, or gabled), including detailed inspection of them with Google Earth, showing results of only automatic procedures and after manual improvement. Finally, a comparison with Google Earth is conducted to assess the outcomes.

There are more possibilities to combine different sources of data. In the work of Qian Zhao et al. [20], LiDAR data are used with information from topographic map Digital Line Graphics (DLGs), where it has been noticed that DLGs and airborne laser point clouds complement each other in providing comprehensive information on the overall 3D structure of buildings.

Creating LoD 2 models under the CityGML standard is a usual base to work with, like the work of Oniga et al. [21], made by taking images with an unmanned aircraft system, creating point clouds and generating the model by various steps of classifications, like if a point is on the ground or not, type of point groups (buildings, vegetation, others), segmentation, etc.

Another work of LoD 2 and CityGML is presented by Drešček et al. [22], who obtained and processed point clouds from unmanned aerial vehicles, including methods to detect patches on the roof faces.

In their paper, B. Xiong et al. leveraged the concept of topology graphs to reconstruct LoD 2 3D building models [23]. Employing a CSG-style approach, they extracted roof edges from point clouds using a foundational element known as the minimum subgraph. Building primitives were then defined based on this, allowing for the representation of any building through a combination of these primitives and subsequent grouping to create the models.

More aspects can be considered for 3D building model generation, such as building types classified in the work of Park and Guildmann [24], who used a trained random forest classifier with a LiDAR point cloud as input. That work classifies the building between commercial, residential, skyscraper, and small constructions.

### 1.1. OpenStreetMap

OpenStreetMap is a collaborative project to create a free and editable world map. Founded in 2004, it is made by volunteers who collect and contribute data about roads, buildings, and other features in their local area. It is a data source and research subject frequently engaged with by the academic community, acknowledging its complex and contextual nature [25].

The resultant map is available for free use and download, offering an alternative to conventional proprietary maps. OSM employs a topological data structure, with four core elements (also known as data primitives):

- *Nodes* are points with a geographic position, stored as coordinates (pairs of numbers, a latitude, and a longitude) according to World Geodetic System (WGS) 84.
- *Ways* consist of ordered lists of nodes, representing a polyline, or potentially a polygon if they constitute a closed loop. They serve the purpose of representing both linear features, such as streets and rivers, and areas, including forests, parks, parking zones, and lakes.
- *Relations* are ordered lists of nodes, ways, and relations (together called “members”), where each member can optionally have a “role” (a string). Relations are used to represent the relationship of existing nodes and ways.
- *Tags* are key–value pairs (both arbitrary strings). They are used to store metadata about the map objects (such as their type, their name, and their physical properties). Tags are not independent entities; instead, they are invariably associated with an object, be it a node, a way, or a relation.

The buildings are the key elements in this work, where each building is a way or *relation* of 2D coordinates (*way* for buildings with single-polygon geometry and *relation* for composition); any other detail like building height is noted as *tags* attached, and the most important of them in this work are tagged “roof:shape”.

### 1.2. Hypothesis

**Figure 1:**
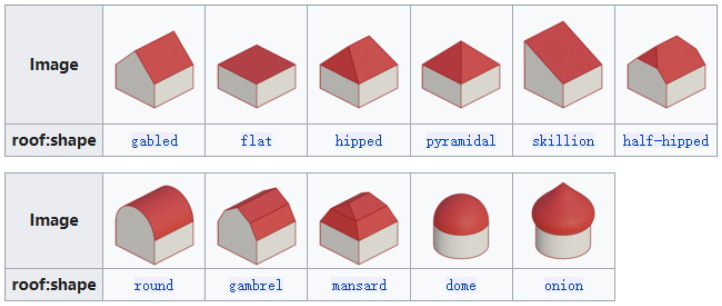
Common roof shapes [26].

This paper proposes a method to add the roofs to polyhedral 3D building models, applicable to buildings with any *node* geometry and any well-defined shape of roof (shown in Figure 1), including some estimation ideas for missing information. Progressively, the following questions will be reviewed in this research:

- Whether a method that uses exclusively OSM information can be created to add the roofs to 3D building models.
- Whether the method can sustain its functionality under certain deviations in the given information.
- Whether the method can sustain its functionality for high-complexity inputs, like buildings with too many sides and buildings with holes.
- Whether the names of shapes of the building roofs can always be distinguishable in the method results.
- Whether the output models can achieve considerable similarity to the actual buildings.

The remainder of this paper is structured as follows: Section 2 provides a comprehensive explanation of the methods employed for reconstructing 3D roofs, particularly focusing on scenarios where roofs possess well-defined shapes. This section is subdivided to address buildings delineated by rectangular and non-rectangular 2D ground coordinates. It elucidates the integration of these methodologies within the overarching process for generating 3D building models. Section 3 presents the outcomes yielded by these methodologies, juxtaposed against outputs generated by online services offering analogous functionalities. Section 4 synthesizes the accomplishments outlined in this paper. Finally, Section 5 outlines potential avenues for future research and development.

## 2. Materials and Methods

In this section, the method used to generate the 3D models is described in detail, where the following information from OSM is used:

- Two-dimensional coordinates of their body.
- Name of roof shape, with possible subparameters.
- Total height of the building.
- Height of the roof part (without the body).

For the 3D modeling, the distance unit must be the same for all the dimensions, and in OSM, the heights are in meters, but the coordinates are in latitudes and longitudes (“WGS84” coordinate system). The solution to this problem is a conversion of the coordinates to the “EPSG:4326” system, which uses meters as a geographic unit; the conversion can be made easily using the tool of *PROJ version 2.8.0* [27].

The algorithms used for roof reconstruction are described separately in full mathematical descriptions and programming considerations. This work adds more details to the theory applied to reconstruct the buildings in the previous research [28].

**Figure 2:**
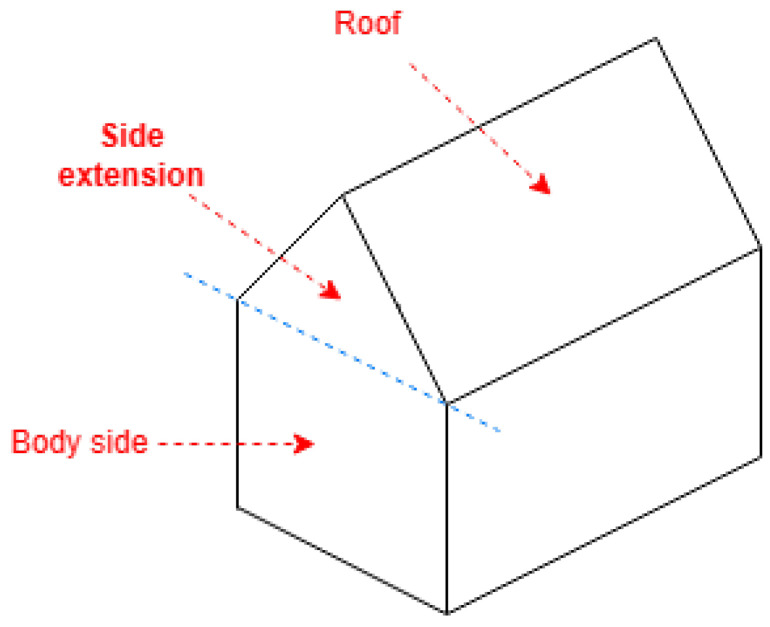
Faces classification.

These algorithms result in 3D models with the faces distinguished between roof and side faces as in Figure 2. This distinction facilitates the assignment of distinct attributes, such as the color of the building, to these respective faces.

The descriptions commence with the most straightforward algorithms designed for buildings with simple rectangular footprints. Subsequently, more intricate algorithms tailored for complex footprints are presented. Additionally, supplementary programming enhancements are explained. Finally, a concise overview of the run-time steps of the ultimate program is provided.

### 2.1. Mathematical Description for Buildings with Simple Rectangle Footprint

A building footprint is considered a simple rectangle that exhibits precisely four exterior sides, with edge angles close to 90º (typically, in the given information, the coordinates are not perfectly perpendicular) and without interior holes.

In the previous research by the same authors [28], the method used to reconstruct the buildings was adapted to be valid for any footprint. This work will process independently these buildings with a simple footprint with an easier theory.

The heights for different vertices on the roof are derived using the heights provided in the OSM information. This process is analogous to the approach outlined in Equation (1) for all buildings with rectangular footprints. However, various roof shapes may need different height calculations.

(1) $$h_{top}=h_{total}\;h_{base}=h_{total}-h_{roof}$$

**Figure 3:**
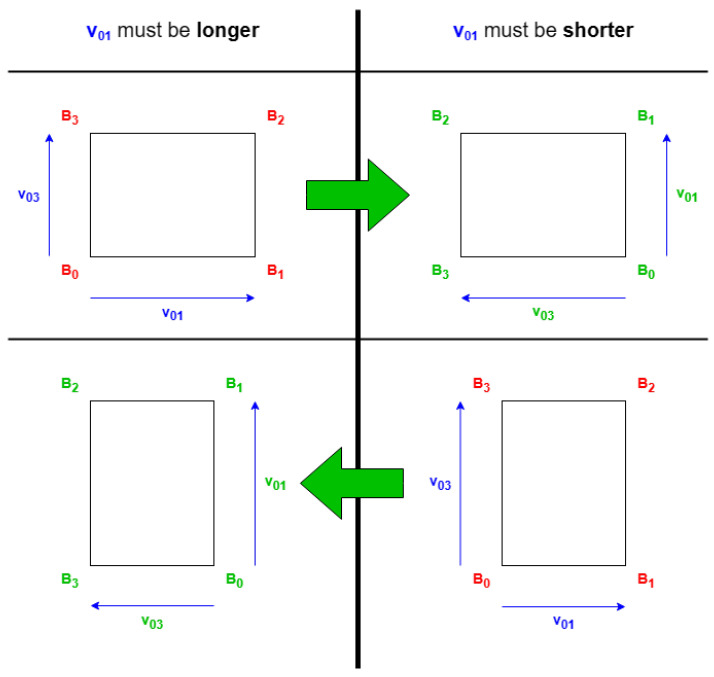
Shift point order for algorithm requirements.

The following algorithms locate most of the upper coordinates on the roof near the first side (between the first two coordinates) and its corresponding opposite side. To meet the concept of roof shapes, these algorithms require that the first side be one of either the shorter or the longer sides, contingent upon the shape and probable properties. To satisfy this, if the provided coordinates are not arranged in a manner that conforms to this requirement, a positional shift can be performed on the coordinates. This shift involves treating the first point as the last and the second as the first (see Figure 3).

#### 2.1.1. Roof Shapes That Do Not Need Side Length Comparison

**Figure 4:**
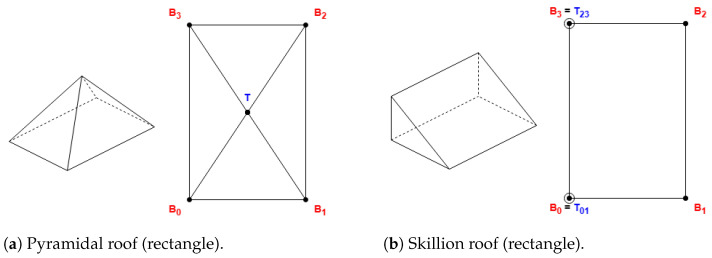
Example of shapes that do not need side length comparison.

Some roof shapes do not require a comparison of side lengths, as the skillion shape displayed in Figure 4, with the definitions in Table 1.

In Table 1, the points $B_{i}$ represent XY coordinates for the base points given in OSM coordinates, being the body side of the building. The points *T* and $T_{ij}$ are the top points, with only XY coordinates. A dot over an uppercase letter means a 3D point with the same XY coordinates as the corresponding 2D point and the proper height of the base (for $B_{i}$) or top (for *T* or $T_{ij}$) up to Equation (1).

The buildings with skillion roofs may have a “direction” parameter: the angle degree from the lower side to the higher. However, in most cases, this information is missing. In most of the examples found where the direction parameter was not given, the second side ($\bar{{\dot{B}}_{1}{\dot{B}}_{2}}$) is the lower, and the last side ($\bar{{\dot{B}}_{3}{\dot{B}}_{0}}$) is the upper.

#### 2.1.2. Roof Shapes That Always Require the First Side to Be Shorter

**Figure 5:**
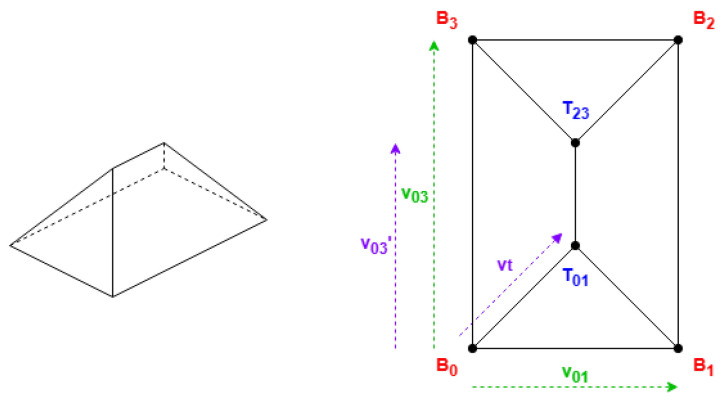
Example of hipped roof with “along” orientation (rectangle).

**Figure 6:**
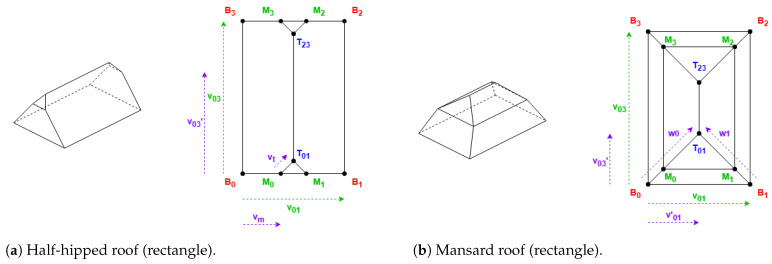
Example of shapes that always require the first side to be shorter.

These roof shapes are in Figure 5 and Figure 6, where the shapes in Figure 6 are derived from Figure 5, with all the definitions in Table 2.

**Figure 7:**
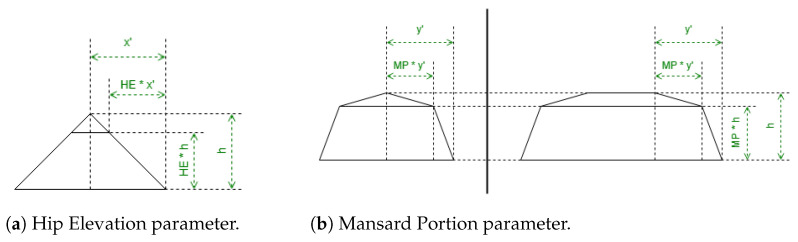
Algorithm parameters.

In Table 2, there are used the parameters Hip Elevation (HE) and Mansard Portion (MP); these are algorithm parameters defined for the half-hipped and mansard shapes, respectively. Their visualizations are displayed in Figure 7.

HE determines the height fraction of hips on the shorter sides to be elevated. Its value must be between 0 and 1, typically greater than 0.5. When HE is equal to 0, the result will be a hipped roof; when HE is equal to 1, the result will be equal to a gabled “along” roof. The recommended value is 0.7.

MP determines the length fraction in the coordinates where the raised edge should be situated on all the hips. Its value must be between 0.5 and 1. When MP is equal to 0.5, the result will be a hipped roof; when MP is equal to 1, the result will be equal to a flat roof. The recommended value is 0.7.

There are also some points $M_{i}$ in Table 2 representing vertices with middle height; the height for these vertices is obtained by Equation (2):

(2) $$\begin{array}{cc} h_{middle}=h_{total}-h_{roof}\cdot \left(1-HE\right) & \\ h_{middle}=h_{total}-h_{roof}\cdot \left(1-MP\right) & \end{array}$$

#### 2.1.3. Roof Shapes That Depend on an OSM Parameter

**Figure 8:**
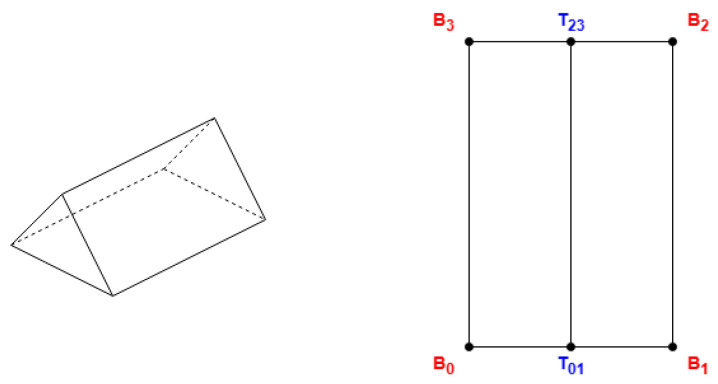
Example of gabled roof (rectangle, along).

**Figure 9:**
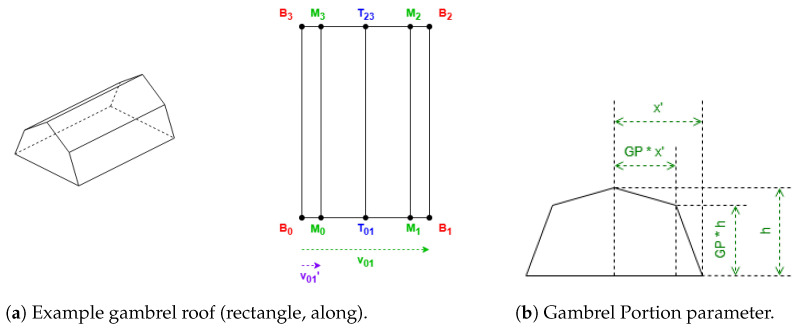
Gambrel roof.

These roof shapes in Figure 8 and Figure 9a have a property “orientation”. Its value can be “along” or “across”. When it is “along”, the algorithm requires the first side to be shorter, and when it is “across” to be longer.

An algorithm parameter Gambrel Portion (GP) is used for the gambrel roof (Figure 9b), which determines the length fraction in the coordinates where the raised edge should be situated on both hips. Its value must be between 0.5 and 1. When GP is equal to 0.5, the result will be equal to a gabled roof; when GP is equal to 1, the result will be equal to a flat roof. The recommended value is 0.7.

The gambrel roof also has vertices with middle height, obtained by Equation (3):

(3) $$h_{middle}=h_{total}-h_{roof}\cdot \left(1-GP\right)$$

When the “orientation” property is not given, the default value will be “along” for gabled roofs and “across” for gambrel. The rest of the definitions of the roof shapes in Figure 8 and Figure 9 are obtained in Table 3.

#### 2.1.4. Example

This theoretical example uses simple values, but the real numbers in practice can have many more integers and floating digits:

- Corners: (125,175),(137,191),(125,200),(113,184).
- Total height: 10.
- Roof height: 2.
- Shape: *gambrel*.
- Orientation: *along*.

First, check the side length requirement, where a gambrel along roof requires the first side to be the shortest.

$$\begin{array}{cc} |\vec{P_{0}P_{1}}|= & \sqrt{{\left(191-175\right)}^{2}+{\left(137-125\right)}^{2}}=20\\ |\vec{P_{0}P_{3}}|= & \sqrt{{\left(184-175\right)}^{2}+{\left(113-125\right)}^{2}}=15 \end{array}$$

If the requirement is not accomplished, apply a position shift on

$$B_{0}=\left(137,191\right)\;B_{1}=\left(125,200\right)\;B_{2}=\left(113,184\right)\;B_{3}=\left(125,175\right)$$

Obtain the 2D coordinates for all the points following the algorithm:

$$\begin{array}{c} {\vec{v}}_{01}=\left(125-137,200-191\right)=(-12,9)\\ {\vec{v}}_{m}=\frac{1-GP}{2}\cdot {\vec{v}}_{01}=\frac{1-0.7}{2}\cdot \left(-12,9\right)=(-1.8,1.35)\\ M_{0}=B_{0}+{\vec{v}}_{m}=\left(137,191\right)+\left(-1.8,1.35\right)=\left(135.2,192.35\right)\\ M_{1}=B_{1}-{\vec{v}}_{m}=\left(125,200\right)-\left(-1.8,1.35\right)=(126.8,198.65)\\ M_{2}=B_{2}-{\vec{v}}_{m}=\left(113,184\right)-\left(-1.8,1.35\right)=(114.8,182.65)\\ M_{3}=B_{3}+{\vec{v}}_{m}=\left(125,175\right)+\left(-1.8,1.35\right)=(123.2,176.35)\\ T_{01}=\frac{B_{0}+B_{1}}{2}=\left(\frac{137+125}{2},\frac{191+200}{2}\right)=(131,195.5)\\ T_{23}=\frac{B_{2}+B_{3}}{2}=\left(\frac{113+125}{2},\frac{184+175}{2}\right)=(119,179.5) \end{array}$$

Finally, obtain and assign the heights to the points:

$$\begin{array}{c} h_{base}=h_{total}-h_{roof}=10-2=8\\ h_{middle}=h_{total}-h_{roof}\;\cdot \;(1-GP)=10-2\cdot (1-0.7)=9.4\\ h_{top}=h_{total}=10 \end{array}$$

### 2.2. Programming Details for Buildings with Rectangle Footprint

All the 3D models are defined by triangulation with their normal vector. In the previous algorithms, the normal vectors of any shape always depend on the ordering of the points following the right-hand rule, in other words, in counterclockwise order from the top view.

Some improvements are applied exclusively for programming the preview algorithms.

Most of the triangulation of the polygons is made by the Node library *earcut* [29]. Specifically, in the case of the quadrilateral roof faces, they can be considered directly as two triangles of $\left(B_{0},B_{1},B_{2}\right)$ and $\left(B_{0},B_{2},B_{3}\right)$ because they can never contain reflex angles, and the *earcut* library cannot triangulate the side faces due to its implementation based on two dimensions.

Some roof shapes have similar aspects to take into account when coding the algorithms:

- For the roof shapes gabled, hipped, and skillion, the upper part of the building has four faces with the same point names. So, those faces can be built with the same function, then differ between roof and side extension faces for each shape.
- Similarly, for the roof shapes half-hipped, gambrel, and mansard, there are eight faces instead, also sharing the point names. Multiple triangles or quadrilaterals can replace the polygons with more sides, maintaining the size of 3D render content (number of vertices and triangles). Following this aspect, the roof faces for these three shapes can be built using the definition for mansard roofs in Table 2.

The footprints are not required to be perfectly perpendicular to qualify as rectangular. To be classified as a rectangle, the scalar product of the unitary vector of two connected sides must have an absolute value less than or equal to 0.01.

### 2.3. Mathematical Description for Buildings with No Rectangle Footprint

**Figure 10:**
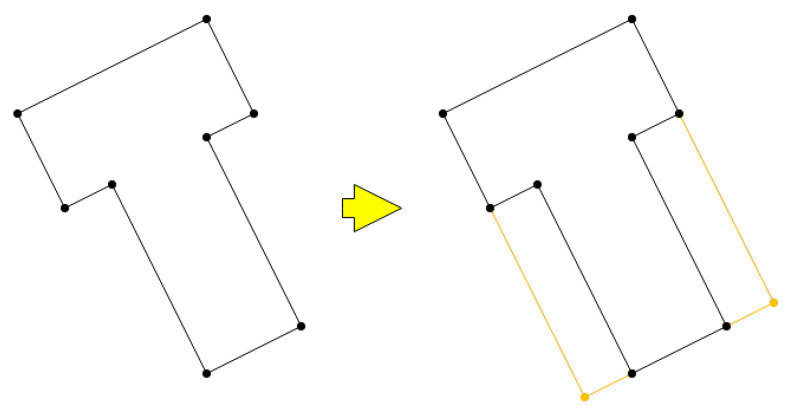
Virtual rectangle roof.

The applied technique involves determining the smallest rectangle area containing all the base coordinates, treating it as a virtual rectangle roof (see Figure 10). All coordinates are then assigned the same height as the corresponding point on this virtual roof.

The 2D coordinates of each roof face depend on the footprint coordinates and on the virtual faces. Existing efficient algorithms for 2D polygons’ intersection or difference [30] can be used in their obtaining processes.

The heights are obtained by finding the formula to translate the coordinates of each building to one or more alternative translated coordinates, then calculating the height by using this alternative value in the specific descending level function (DLF) defined for each shape of the roof.

#### 2.3.1. Alternative Translated Coordinates

First, the alternative coordinates should have their axis parallel to the longest or second longest direction of sides of the building, also referred to as the primary or secondary side direction.

These directions are chosen between the vector orientations of the outer ring of the footprint coordinates. The vectors can be grouped if their directions are identical or opposite. Their behavior will be the same. Considering possible deviations in the given information, the grouping of two vectors is evaluated by whether the scalar product of their unitary vector is more than 0.99 or less than −0.99.

In each group, the next step is to sum or subtract the length of each vector in the same order as the coordinates, depending on whether the directions are opposite or not, storing the maximum and minimum values in the steps of the cumulative sum. The difference between those values is the length of this direction.

The values in the alternative coordinates need the following properties:

- Proportional with the distance to the alternative axis.
- Within [−1, 1], required by the DLFs.

For the first property, the alternative value is obtained by the value of a scalar product of the position vector between points of each original coordinate and the normal vector to the alternative axis.

Then, with the maximum and minimum in the previous values, we reflect them to the range of [−1, 1].

In conclusion, to translate a coordinate, the normal vector to the alternative axis and the following parameters are required considering all the exterior original coordinates:

(4) $$\begin{array}{c} x_{0}^{\prime }=\frac{max\left({\vec{n}}_{x}\cdot x_{i}+{\vec{n}}_{y}\cdot y_{i}\right)+min\left({\vec{n}}_{x}\cdot x_{i}+{\vec{n}}_{y}\cdot y_{i}\right)}{2}\\ reduction_{x^{\prime }}=\frac{max\left({\vec{n}}_{x}\cdot x+{\vec{n}}_{y}\cdot y_{i}\right)-min\left({\vec{n}}_{x}\cdot x_{i}+{\vec{n}}_{y}\cdot y_{i}\right)}{2} \end{array}$$

The maximum and minimum of the alternative value are computed on a set with all exterior points $\left(x_{i},y_{i}\right)$. With it, the alternative value for any point (x,y) is obtained by

(5) $$x^{\prime }=\frac{{\vec{n}}_{x}\cdot x+{\vec{n}}_{y}\cdot y-x_{0}^{\prime }}{reduction_{x^{\prime }}}$$

**Figure 11:**
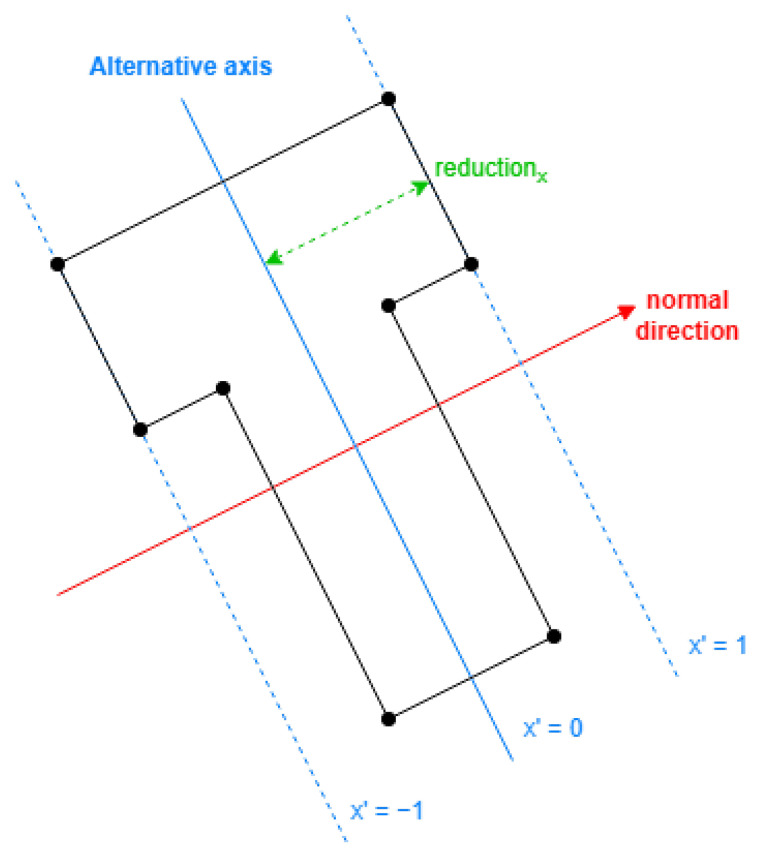
Example of alternative coordinate in a direction.

As displayed in Figure 11, the normal direction is selected first, then the parameters can be obtained with Equation (4). The resulting definition will reflect the coordinates using Equation (5).

In this document, when the double alternative axis is required, the coordinate $x^{\prime }$ will always be longer than $y^{\prime }$. The coordinate $x^{\prime }$ will be based on the secondary side direction, where the alternative axis is parallel to it, having a bigger value in the reduction parameter. Furthermore, $y^{\prime }$ will be based on the primary.

#### 2.3.2. Alternative Face Positions

By employing two alternative coordinates, the coordinate translation can be reverted. For each roof shape, there exist specific static alternative 2D positions that facilitate the construction of roof faces. These positions can be reversed and translated back to the actual coordinates.

For example, given two alternative coordinates:

(6) $$x^{\prime }=\frac{{\vec{n}}_{x}\cdot x+{\vec{n}}_{y}\cdot y-x_{0}^{\prime }}{reduction_{x^{\prime }}}\;y^{\prime }=\frac{{\vec{m}}_{x}\cdot x+{\vec{m}}_{y}\cdot y-y_{0}^{\prime }}{reduction_{y^{\prime }}}$$

To obtain a point which requires the alternative values $\left(x_{1}^{\prime },y_{1}^{\prime }\right)$, we use a simple linear equation system with two unknowns:

(7) $$\left\{\begin{array}{c} {\vec{n}}_{x}\cdot P_{x}+{\vec{n}}_{y}\cdot P_{y}=x_{1}^{\prime }\cdot reduction_{x^{\prime }}+x_{0}^{\prime }\\ {\vec{m}}_{x}\cdot P_{x}+{\vec{m}}_{y}\cdot P_{y}=y_{1}^{\prime }\cdot reduction_{y^{\prime }}+y_{0}^{\prime } \end{array}\right.$$

Furthermore, the point will be defined by values $\left(P_{x},P_{y}\right)$.

#### 2.3.3. Face Cross Points

**Figure 12:**
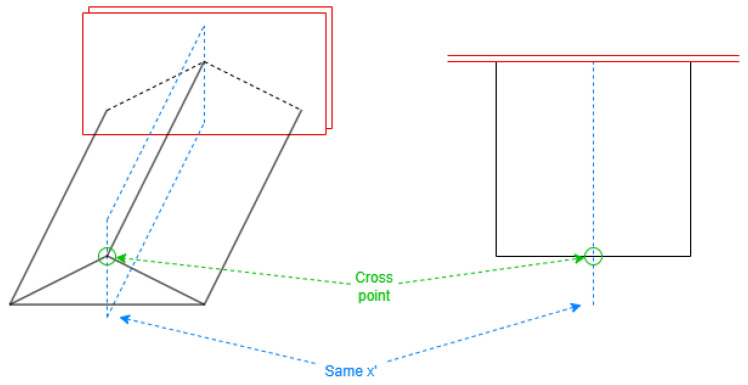
Cross point.

When a 2D side is part of the perimeter for more than one 3D face, those coordinates where different faces cross must be considered. These are vertices on top to build the faces of the roof and side extension but not included in the footprint coordinates (Figure 12).

This process is vital to properly build the cases like the bottom triangle face in the left part of Figure 12 to create closed volumes. It must be performed with the coordinates of the exterior sides and all the holes, iterating each segment. A triangle or quadrilateral side extension face will be created from each side of the building to the roof height for the points.

When a side contains many cross points, they are ordered by the scale applied to the side vector.

##### Single Alternative Axis

**Figure 13:**
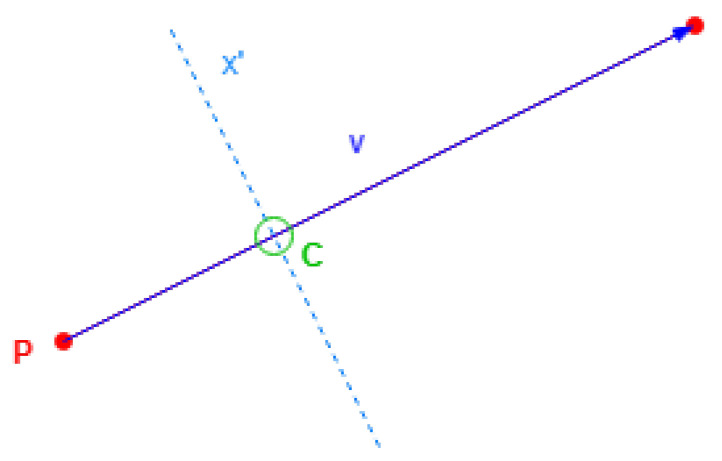
Cross value for single alternative axis.

The cross points appear in specific lines with constant values in the single alternative coordinate, called *cross values*, regarding Figure 13. The value of 0 is commonly employed, typically representing the line with the maximum height on the building roof.

Starting with Equation (8), a cross point *C* is obtained by dragging a building coordinate point *P* by a scale of the side vector $\vec{v}$ to have a determinate *cross value* $x_{C}^{\prime }$:

(8) $$C=P+scale\cdot \vec{v}\;\frac{{\vec{n}}_{x}\cdot C_{x}+{\vec{n}}_{y}\cdot C_{y}-x_{0}^{\prime }}{reduction_{x^{\prime }}}=x_{C}^{\prime }$$

The only thing required is for this scale to be dragged, which can be obtained by uniting the preview equations and obtain scale alone.

Replacing *C* to obtain Equation (9):

(9) $$\frac{{\vec{n}}_{x}\cdot \left(P_{x}+scale\cdot {\vec{v}}_{x}\right)+{\vec{n}}_{y}\cdot \left(P_{y}+scale\cdot {\vec{v}}_{y}\right)-x_{0}^{\prime }}{reduction_{x^{\prime }}}=x_{C}^{\prime }$$

The value of scale will be as in Equation (10):

(10) $$scale=\frac{x_{C}^{\prime }\cdot reduction_{x^{\prime }}-{\vec{n}}_{x}\cdot P_{x}-{\vec{n}}_{y}\cdot P_{y}+x_{0}^{\prime }}{{\vec{n}}_{x}\cdot {\vec{v}}_{x}-{\vec{n}}_{y}\cdot {\vec{v}}_{y}}$$

If a building side has a cross point with an alternative position value, the scale obtained must be within range (0, 1), with the limits excluded to avoid point repetitions.

These points are also used further in Section 2.3.5.

##### Double Alternative Axis

**Figure 14:**
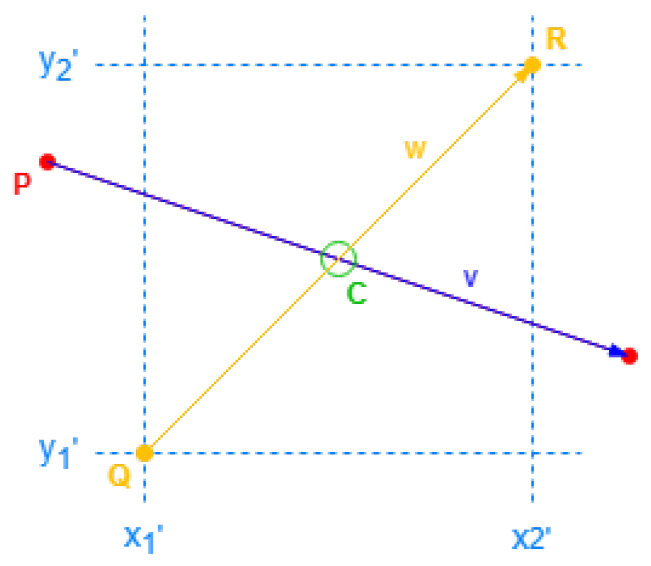
Intersection of cross segments.

For the double alternative axis, the cross points are intersections of building sides and specific segments on the roof. Those *cross segments* are the inner segments existing in the virtual rectangle roof, and they can be obtained by a reverse translation from Alternative Face Positions defined for the shape of the roof. The cross points are obtained with these segments according to Figure 14.

Considering the building coordinate *P* and the side vector $\vec{v}$, and a *cross segment* with the format of starting point *Q* and the vector $\vec{w}$, a cross point is defined by

(11) $$C=P+scale_{v}\cdot \vec{v}=Q+scale_{w}\cdot \vec{w}$$

The scales can be resolved by a linear equation system with two unknowns:

(12) $$\left\{\begin{array}{c} {\vec{v}}_{x}\cdot scale_{v}-{\vec{w}}_{x}\cdot scale_{w}=Q_{x}-P_{x}\\ {\vec{v}}_{y}\cdot scale_{v}-{\vec{w}}_{y}\cdot scale_{w}=Q_{y}-P_{y} \end{array}\right.$$

If a side intersects with a cross segment, both scales need to be within range (0, 1), where $scale_{v}$ has the limits excluded to avoid point repetitions, and $scale_{w}$ has the limits included. Furthermore, $scale_{v}$ is used to order many cross points on the same side.

#### 2.3.4. Descending Level Function

OSM information provides the total height of the entire building and the height of the roof part only. The difference between those values is the minimum height of all the points on the roof. The height value of each point on the roof can be obtained by descending a certain distance from the total height value. This distance is a portion of the height of the roof part, related to values in the alternative coordinates.

The points on flat roofs do not need this kind of descent. However, for other shapes, a DLF will be assigned to each necessary alternative coordinate to obtain the descending portion of each point. In cases where two alternative coordinates are utilized, the final descending portion will involve taking the maximum value between them.

(13) $$\begin{array}{c} h\left(x^{\prime }\right)=h_{total}-h_{roof}\cdot DLF\left(x^{\prime }\right)\\ h\left(x^{\prime },y^{\prime }\right)=h_{total}-h_{roof}\cdot max\left(DLF_{x}\left(x^{\prime }\right),DLF_{y}\left(y^{\prime }\right)\right) \end{array}$$

#### 2.3.5. Processing Steps for Roof Shapes Using a Single Alternative Axis

**Figure 15:**
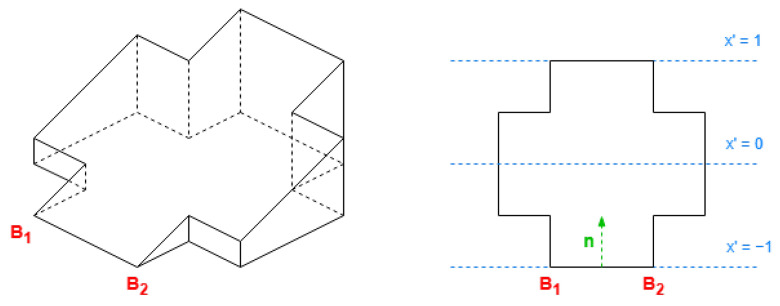
Example of skillion roof (not rectangle).

**Figure 16:**
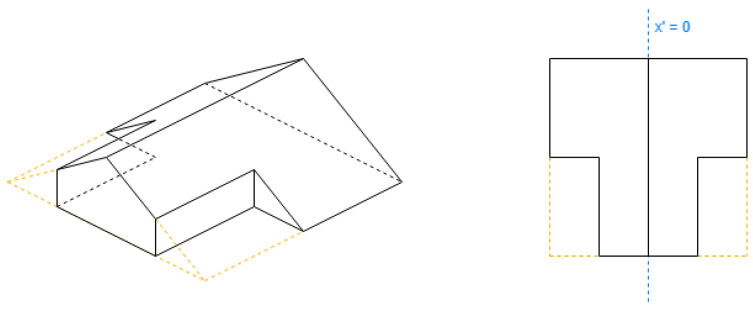
Example of gabled roof (not rectangle).

**Figure 17:**
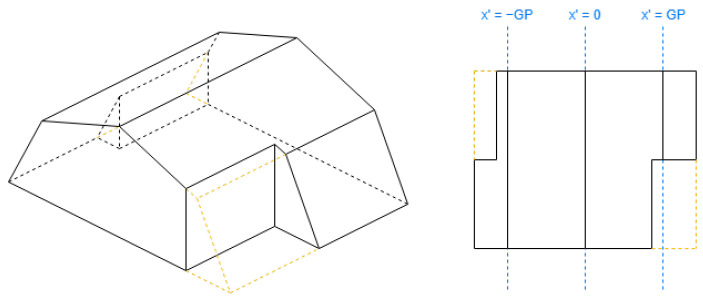
Example of gambrel roof (not rectangle).

These shapes are skillion (Figure 15), gabled (Figure 16), and gambrel (Figure 17).

Again, skillion roofs may have a “direction” parameter missing in most cases. When this parameter is given, it will be used as the normal vector to the alternative axis. Otherwise, the second side ($\bar{{\dot{B}}_{1}{\dot{B}}_{2}}$) will be considered as the lower, whose normal vector will be also normal to the alternative axis, as in Figure 15.

Furthermore, for the “orientation” property for gabled and gambrel roofs, when it is “along”, the alternative axis will be parallel to the primary side direction, when it is “across”, the secondary.

Using the cross values defined in Table 4, the base coordinates with the cross points are added following Section 2.3.3. Then, a distribution can be made to the coordinates at the exterior sides to obtain the inclined faces. This distribution follows the order of the information given. Each inclined face is defined by the ranges of alternative values divided by the *cross values*.

For example, in Figure 17, there are 3 *cross values*, and the roof will have 3 + 1 ranges or inclined faces, where every cross point becomes a vertex for two roof faces.

With each face formed, the next step is to obtain the crop area difference of each face with all the holes. Existing algorithms can be used to achieve that [30]. The obtained polygons from the difference will be the 2D coordinates of the final roof.

Finally, the DLF in Table 4 can obtain the vertex heights to create the roof volumes.

#### 2.3.6. Processing Steps for Roof Shapes Using the Double Alternative Axis

**Figure 18:**
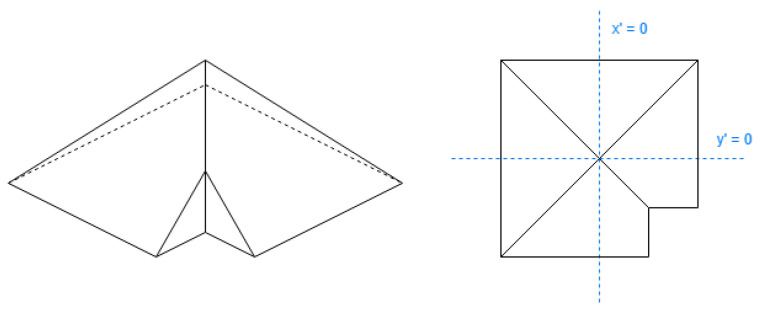
Example of pyramidal roof (not rectangle).

**Figure 19:**
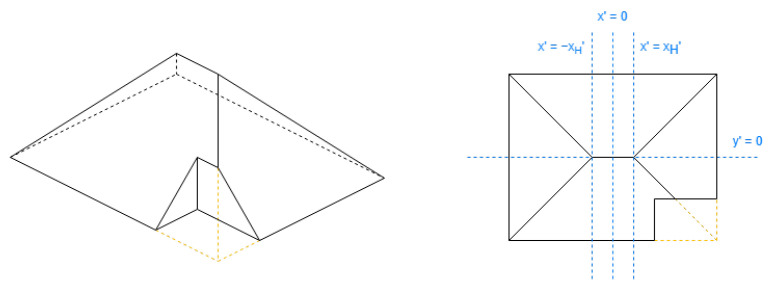
Example of hipped roof (not rectangle).

**Figure 20:**
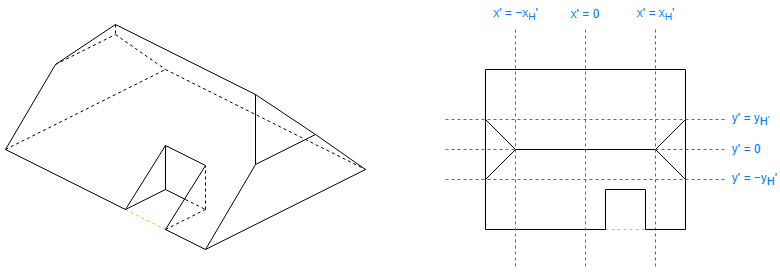
Example of half-hipped roof (not rectangle).

**Figure 21:**
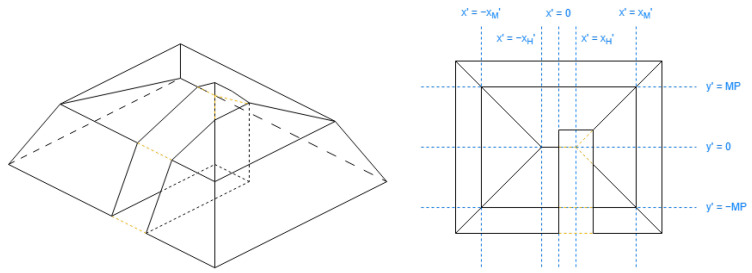
Example of mansard roof (not rectangle).

These shapes are pyramidal (Figure 18), hipped (Figure 19), half-hipped (Figure 20), and mansard (Figure 21).

The roof generation is based on the virtual rectangle roof, whose coordinates can be obtained by reverse translation from these Alternative Face Positions.

The definition of many roof shapes contains special positions on the alternative coordinates, listed in Table 5, mostly depending on the scale between the extension length of both alternative coordinates ($reduction_{x^{\prime }}$ and $reduction_{y^{\prime }}$) for each building. The equations are independent for each roof shape. These geometries on the alternative coordinates based on these special positions are listed in Table 6.

The value of $x_{H}^{\prime }$ for half-hipped roofs in Table 5 may be understood better with this expression in Equation (14), comparing Figure 19 and Figure 20:

(14) $$\left(1-\frac{reduction_{y^{\prime }}}{reduction_{x^{\prime }}}\right)+HE\cdot \frac{reduction_{y^{\prime }}}{reduction_{x^{\prime }}}$$

The cross points can be obtained following Section 2.3.3 using the cross segments in Table 6, and these points construct the segments containing the side extension faces to close the volume.

The 2D coordinates of the resulting roof faces are obtained by 2D intersections [30] of the building footprint coordinates with each virtual face in Table 6.

The half-hipped and mansard shapes are variants of the hipped shape, representing a shared aspect. Regarding Table 5, the special value $x_{H}^{\prime }$ is obtained to discard a middle section on the longest side coordinate; it will be reflected further with the DLFs.

The mansard roof can be viewed as a hipped roof with two slopes on each side, their positioning dependent on the algorithm parameter of MP. The list of cross segments for the mansard shape contains all the segments for the hipped shape.

Finally, the height of the vertices is obtained using the DLFs in Table 7 to create all the roof faces and side extension faces. Applying Equation (13), the descent height portion for each vertex will be the maximum between the results of both DLFs.

Note in Table 7, $DLF_{x}$ depends on $DLF_{y}$ for the mansard roof shape.

### 2.4. Compatible Additional Developments

#### 2.4.1. Output Model Formats

Initially, the existing literature relies on Wavefront OBJ files as the exclusive output format, which has certain limitations. The data are stored in a human-readable plain-text format and are only suitable for static models.

An option of using the Graphics Library Binary (GLB, binary version of Graphics Library Transmission Format) output format has been added in this work, which is computationally more efficient by binary storage and allows real-time updates on the models for web usage.

#### 2.4.2. Adjustments to Radio-Propagation Requirements

This enhancement is specific to the GLB format. In radio-propagation operations, if a ray falls within the gap between two connected buildings, it necessitates a mirror reflection. However, the actual reflection may experience deviations around the corners.

To resolve this problem, the solution is to consider the connected buildings or 3D models as unique ones, with support of the Node version of library *THREE-CSGMesh* [31], applying geometry unions to the connected models. When dealing with a sufficiently large number of buildings, the geometry union process can become time-consuming. Therefore, its application should be presented as an option that users can decide upon through the user interface.

#### 2.4.3. Special Roof Shapes

The following roofs have no details to be considered for exclusive math description, but specific details for programming exist for them. When a building has those roof shapes, the same programmed algorithms are used for rectangle and non-rectangle roofs, based on alternative coordinates used for non-rectangle footprints (Section 2.3.1).

##### Round Roof

In 3D modeling, any circular shape is represented by a collection of triangles, and the round roofs are half cylinders for the buildings with rectangle footprints. So, the algorithm requires a parameter about how many faces should be used for the round roof for this approximation.

A single alternative axis is required, which is based on the primary side direction for roofs with orientation “along” and based on the secondary side direction for orientation “across”.

The *cross values* depend on the number of faces used for approximation to the half cylinder, dividing the range (−1,1) in that number of parts, then use the limit values as the *cross values*, excluding −1 and 1 themselves.

For example, when eight faces are used to resemble the half cylinder, the *cross values* will be

(15) $$x_{C}^{\prime }\in \left\{-0.75,\;-0.5,\;-0.25,\;0,\;0.25,\;0.5,\;0.75\right\}$$

The descending level function is

(16) $$DLF\left(x^{\prime }\right)=1-\cos\left(x^{\prime }\cdot \frac{\pi }{2}\right)$$

##### Onion and Dome Roof

Those roofs with sphere parts are defined mainly by a mesh in the Three. JS library [32], so those roof shapes are exclusive to the GLB format.

Double alternative coordinates are needed to locate the center location of each building, where $x^{\prime }=0\wedge y^{\prime }=0$.

The building body, in isolation, is represented by a flat roof. The final mesh of a building is then created by combining the body with the specialized roof mesh.

A sphere mesh is required for both roof shapes. The XY of the center can be obtained by reverse translation on the alternative coordinates (7), the Z of the center will be the building height, and the sphere radius depends on the shorter coordinate. The value of $reduction_{y^{\prime }}$ from the secondary alternative coordinate found in Equation (4) is the perfect value. With it, the XY scope of the sphere mesh never stands out from the building body.

In the case of a dome roof, an additional cone mesh extending from the preceding sphere mesh is necessary. The base center of the cone also aligns with the center location of the building in the XY position. However, there is no singular solution for the other parameters, the Z position of the cone base center, the base radius, and the cone height. A possible solution is

(17) $$r_{cone}=h_{cone}=\frac{reduction_{y^{\prime }}}{\sqrt{2}}\;B_{z}=h_{base}+r_{cone}\cdot 1.5$$

#### 2.4.4. Missing Data Estimation

Certain information is unavailable from OSM, necessitating the implementation of judicious decisions to complete the data and facilitate the execution of the programmed algorithm. For instance, the essential heights for the foundation of the 3D modeling are tagged on merely 2.9% of the buildings [33].

These are the decisions for qualitative parameters:

- When the roof shape of a building is not given, the roof will be considered flat.
- At times, the classification of roof shape is designated as pitched, encompassing all roof configurations with inclinations. In such cases, these roofs will be construed as gabled given that this category constitutes the predominant type observed in practical instances.
- In instances where a building is characterized by a roof shape classified as gabled, hipped, gambrel, or round yet lacks specific orientation information, the assumed orientation will be deemed as across for gambrel roofs, aligning with the prevalent orientation observed in other roof types. This estimation aligns consistently with all identified examples.

The following determinations pertain to quantitative parameters, specifically the total height of buildings and the height of their roofs. In most cases, these determinations rely on referencing existing data from other buildings within the same request area:

- On occasion, precise height values may not be explicitly provided; however, estimations can be derived based on parameters such as the number of levels in the building, body levels, and roof levels. An assumed average height of 3.5 m per level is considered for these estimations. It is noteworthy that there may be variations in attribute names for these parameters, with pairs like “levels” and “building:levels”, as well as “roofLevels” and “roof:levels”, conveying equivalent meanings.
- When height information is missing in part of the buildings in an area request, those buildings can use average values from the others with values given, including the values obtained in the previous estimation by levels. This estimation proves effective in areas where buildings share similar or identical heights. However, a validation check is imperative for this estimation, ensuring that the height attributed to the roof never surpasses the total height of the building.
- When all the buildings in an area request do not have the building total height given, a default of 8 m will be used.
- In cases where the roof height is not provided for all buildings within a given area request or when values derived from other estimations lack coherence, the roof height will be deemed as one-tenth (1/10) of the total height.

#### 2.4.5. Others Important Parameters

The minimum height is a straightforward parameter for some buildings that do not contact the ground; it involves assigning distinct Z coordinates to the bottom face of the corresponding 3D features.

Another parameter is the building colors. Each building may have a body color and a roof color. In this work, the colors are exclusively tailored for the GLB format, wherein the mesh functionality permits the incorporation of color definitions for each vertex within every triangle. Notably, as the current requirements do not necessitate gradient colors, each triangle is uniformly assigned a single color across all three vertices.

The vertices can be part of many triangles but with different color definitions for each one.

In OSM data, color definitions exclusively vary between roof color and body color. A default gray color will be applied when such definitions are absent.

It is important to note that the ground, for which appropriate definitions are yet to be found, is also rendered with a default gray color.

### 2.5. Final Run-Time Steps Description

**Figure 22:**
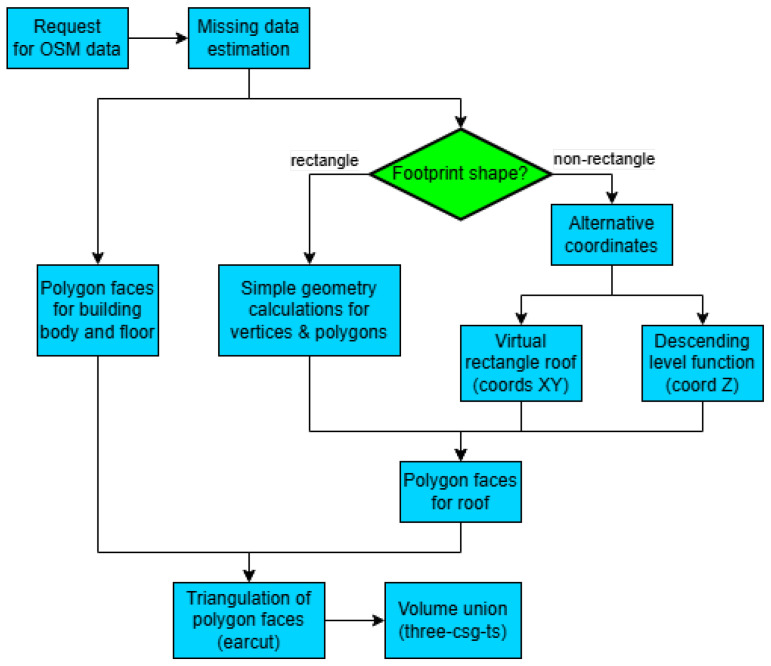
Flowchart of the entire research methodology.

This section gives a quick view of the steps made by the program to build the 3D model of an area (illustrated in Figure 22):

- Send a request to the service of the data mining tool overpass-turbo [3] for a specific range of latitude and longitude and containing only building features.
- Check the information given, filter strange values for known parameters, like a roof shape without an algorithm defined, and try to fill in some missing information as described in Section 2.4.4.
- Process each building feature, create the base building sides by creating a vertical rectangle for each segment of the building 2D coordinates (exterior and holes) and also the bottom face by triangulation of the full coordinates with earcut [29].
- When the roof of the building has a non-flat shape with the algorithm defined, obtain and add the roof and side extension faces to the model. Otherwise, use a single flat top face of full coordinates, making a similar triangulation as in the bottom face.
- Complete the 3D model by union with the mesh functionality if the desired output format supports it.
- Consolidate the various interconnected buildings in 3D space into a unified 3D feature to mitigate radio-propagation deviations.
- Create the output model with merged features, with all 3D coordinates and coloring already defined, and add a ground face at the end.

## 3. Results

This section focuses on comparing images generated by the model with actual building images, with the primary objective being to assess and emphasize their similarity.

The output models from the program were rendered in Microsoft 3D Viewer to take the images, making screen captures of specific sections of the models.

To facilitate this comparison, essential reference points are required. The most expedient source involves acquiring top-view satellite images from Google Maps to assess the similarity of roof shape with real images. Additionally, images from Google Earth, resembling actual photographs, are employed for a thorough comparison. The outcomes are cross-referenced with those obtained from the proprietary 3D viewer of OSM [18].

### 3.1. Experiment Results of Single Buildings

With the supplementary details provided in Section 2.4.4, the generation of 3D building models, inclusive of added roofs, can now be undertaken using solely the information available in OSM.

**Figure 23:**
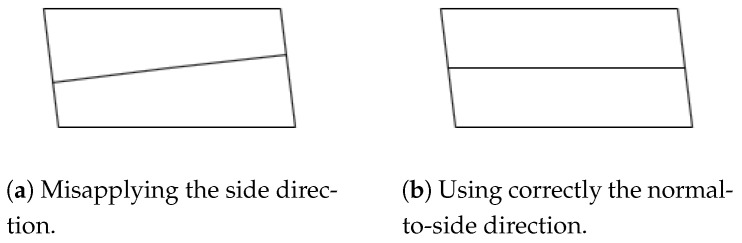
Alternative-coordinate-generated mode comparison.

The primary discrepancy in the OSM data lies in the fact that the majority of right-angled corners in building coordinates deviate from exact 90-degree angles. This is particularly evident in the alternative coordinates discussed in Section 2.3.1. These coordinates are constructed based on the normal direction to the sides rather than aligning with the same direction as the side. This approach ensures that the geometries and models accommodate twisted angles, as illustrated in Figure 23b.

It is necessary to consider the rest of the items of the hypothesis separately for rectangles and non-rectangles.

#### 3.1.1. Simple Buildings with Rectangle Footprint

**Figure 24:**
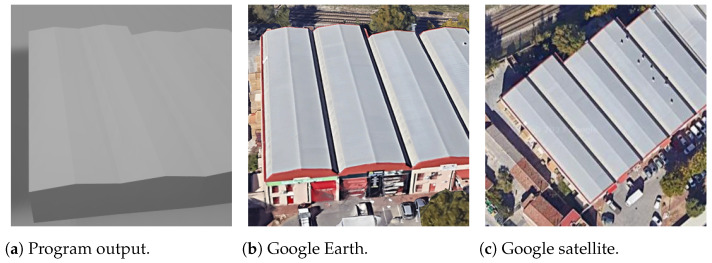
Gabled rectangle building.

**Figure 25:**
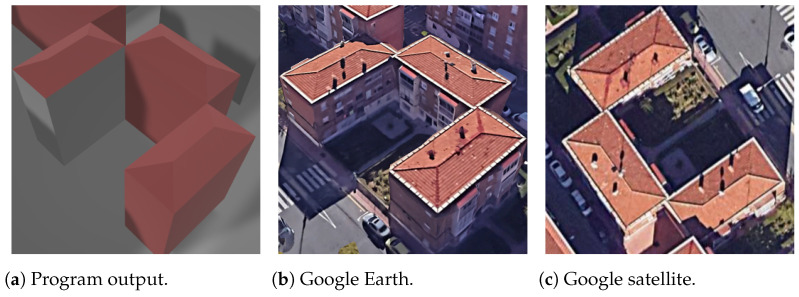
Hipped rectangle building.

First, for the most simple and most common roof shapes of gabled and hipped, shown in Figure 24 and Figure 25, respectively, the vertices of the program output are very well coincident with the reference capture from Google Earth. They also show the aspect of processing the roof faces and side faces separately, having different colors in this case.

**Figure 26:**
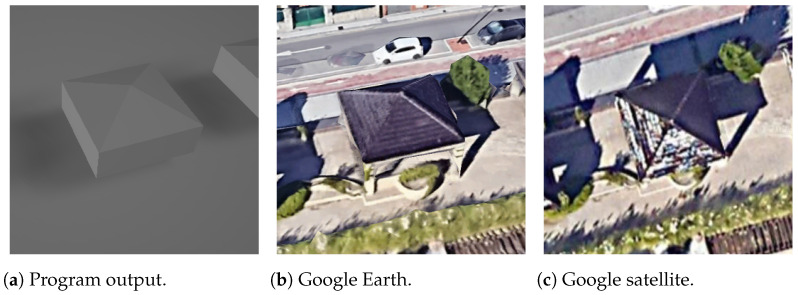
Pyramidal rectangle building.

In Figure 26, depicting a building with a pyramidal roof, there is a notable alignment among the vertices of the roof. However, it is important to note that the windows or gaps on the sides are not within the scope of this work and, therefore, are not considered.

**Figure 27:**
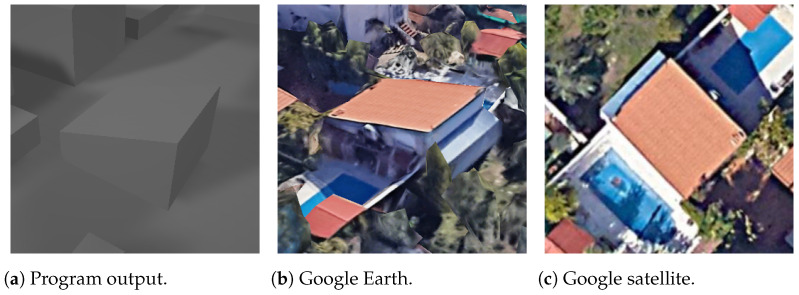
Skillion rectangle building.

As a good case, the lower and upper side positions are correctly predicted for a skillion roof in Figure 27, but it may be false for some other buildings with this roof shape. This is a problem that cannot be solved using only OSM information.

**Figure 28:**
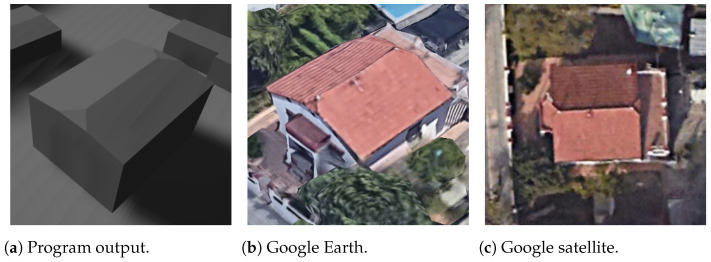
Half-hipped rectangle building.

**Figure 29:**
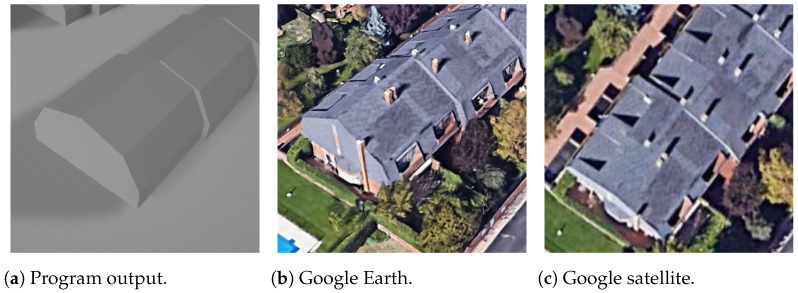
Gambrel rectangle building.

For more intricate roof shapes such as half-hipped and gambrel, the 3D models can still exhibit coincident vertices on the roof, as illustrated by the buildings in Figure 28 and Figure 29. However, it is noteworthy that in some instances, the body of the building appears slightly bloated. This discrepancy arises when the actual 2D coordinates of the building footprint yield an area somewhat smaller than that of the roof. Such cases are not regulated in the OSM information and may not be discernible in satellite images from Google Maps.

**Figure 30:**
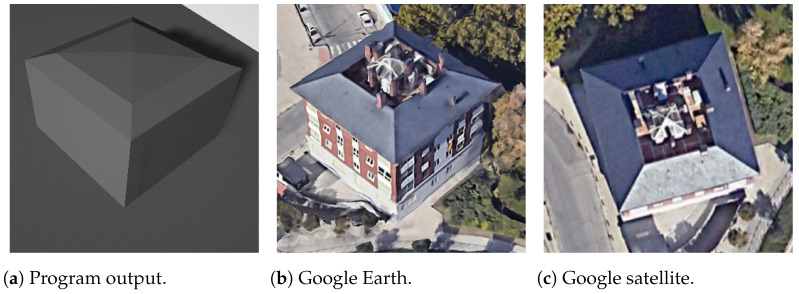
Mansard rectangle building.

Additional aspects of buildings not accounted for in OSM information are exemplified in Figure 30, where a building with a cavity on the roof is depicted. These cavities are not treated as holes in the 2D coordinates provided by OSM, as they do not extend to the ground level. While such details may not significantly impact the overall performance of the building, it is important to acknowledge that they fall outside the scope of control in this context.

The structures with rectangular footprints do not require highly complex inputs. The preceding results demonstrate that all roof shapes are distinguishable, with 3D model vertices exhibiting a striking similarity to their real counterparts.

#### 3.1.2. Complex Buildings

**Figure 31:**
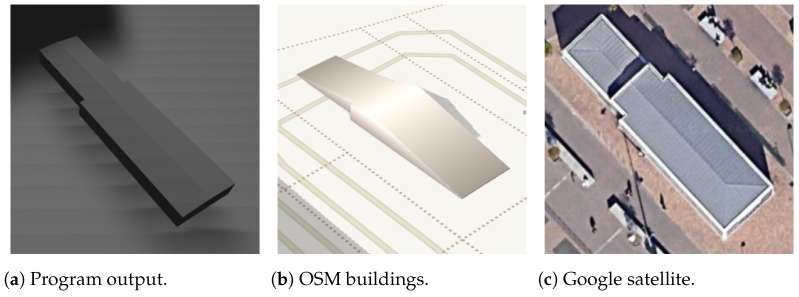
Gabled non-rectangle building.

OSM is susceptible to inaccuracies, as evident in the case of the building showcased in Figure 31, where the assigned roof shape name suggests a gabled roof when, in reality, it should be classified as hipped. Despite such discrepancies, the program output correctly depicts the shape of a hipped roof, demonstrating that the 3D model can accurately represent real buildings even in instances of incorrect information in OSM.

**Figure 32:**
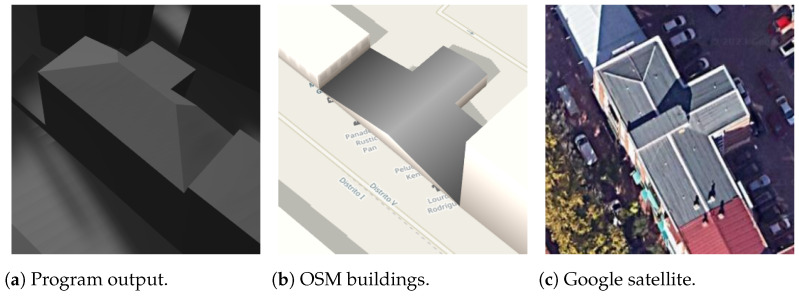
Hipped non-rectangle building (the text in the figures is unrelated to this article).

While OSM information is generally accurate, Figure 32 illustrates a scenario where the program output correctly identifies the roof shape as hipped, yet the vertices do not closely resemble their real counterparts. It is important to acknowledge that the proposed technique of virtual rectangle roofs, as presented in this paper, is suitable for only a subset of buildings. Further developments are needed to address this limitation and enhance the applicability of the method.

**Figure 33:**
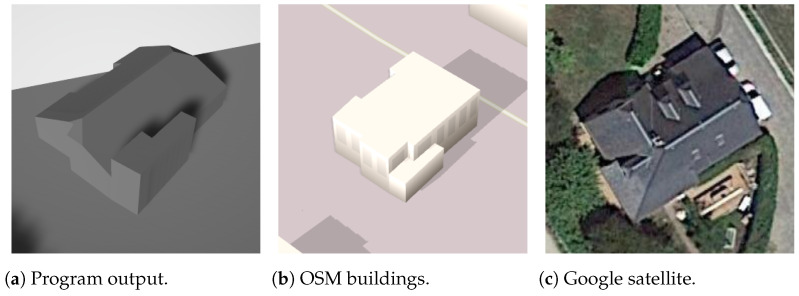
Half-hipped non-rectangle building.

The visual classification of roof shapes relies significantly on the visibility of their edges. In the roof model presented in the program output of Figure 33, a portion of the roof edges within its virtual rectangle roof is not entirely displayed in the final model. Consequently, the shape becomes challenging to discern due to incomplete visibility.

**Figure 34:**
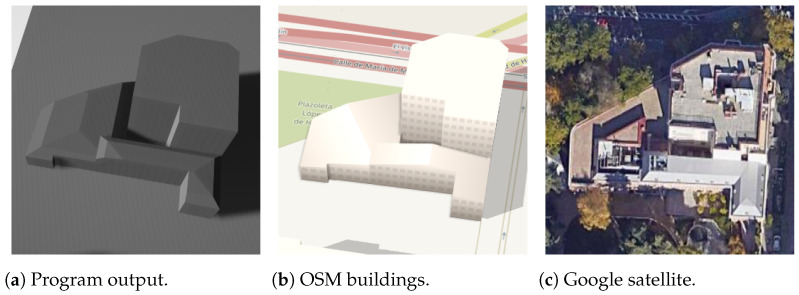
Mansard non-rectangle building (the text in the figures is unrelated to this article).

In Figure 34, which comprises three distinct buildings, one flat and two mansard roofs, the distinguishability of roof shapes is even more challenging compared to Figure 33. Particularly, the building at the bottom right demonstrates a pronounced issue, wherein two critical roof edges on its virtual rectangle roof are entirely omitted in the final 3D model. Consequently, the resultant 3D model fails to closely resemble the real buildings in this case.

While the results for buildings with non-rectangular footprints exhibit a somewhat improved performance compared to OSM buildings [18], the overall similarity to real buildings is not entirely satisfactory. The method proves effective in certain cases but falls short of providing consistently accurate representations for a broader range of buildings.

### 3.2. Experiment Results of Request Areas

**Figure 35:**
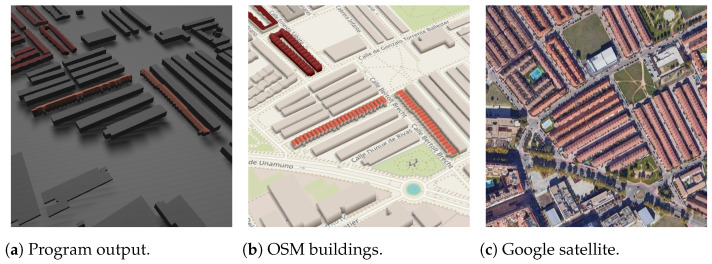
Area example (longitude 40.497527, latitude −3.370850, the text in the figures is unrelated to this article).

Analyzing the visual aspects in Figure 35, several evident issues arise. The satellite image indicates that the majority of the buildings in this area feature light red gabled roofs, yet this information is not adequately reflected in OSM data. Consequently, both the program output in this work and the OSM buildings exhibit disparities in the generated 3D models compared to similar real-world buildings. In all such instances, the quality of the output results is significantly hampered by the incompleteness of information in the datasets.

The gabled across roofs tagged in OSM are accurately reconstructed in the specified area request. This holds true for buildings with both rectangular and non-rectangular footprints. In such cases, the top edge is appropriately positioned between the farthest sides, ensuring a correct representation in the generated 3D models.

The planar buildings situated at the bottom-left corner of the image lack height information and are tagged with only one building level, leading to a height estimation of 3.5 m. This fixed value may not be suitable, as buildings with only one level can vary significantly in height. Future enhancements may involve incorporating additional estimations to more accurately capture the diverse heights of single-level structures.

**Figure 36:**
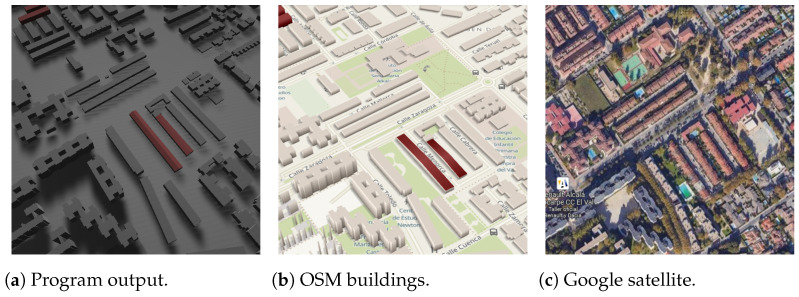
Area example (longitude 40.486543, latitude −3.343740, the text in the figures is unrelated to this article).

The results in Figure 36 also have the problem that only a small portion has the roof shape defined.

## 4. Discussion

This section discusses the achievement of the method and its results concerning the hypothesis. No studies with the same focus were found for comparison.

In this paper, the algorithms to add roofs to 3D building models using OSM information were created. Regarding the first hypothesis, “whether it is possible to create a method to add the roofs to 3D building models using exclusively OSM information”, the possibility was successfully demonstrated by combining the algorithms up to the aspect parameters.

Regarding the second point, “whether the method can sustain its functionality under certain deviations in the given information”, the algorithms maintained their functionality despite certain deviations in the provided information, particularly in cases of missing data.

Concerning the third hypothesis, “whether the method can sustain its functionality for high-complexity inputs, like buildings with too many sides and/or holes”, the algorithms exhibit excellent precision for buildings with rectangular footprints. However, for structures with complex footprints, the method performs well only when the buildings have holes and exterior borders similar to rectangles.

Regarding the fourth hypothesis, “whether the names of shapes of the building roofs can always be distinguishable in the method results”, two challenges were identified. Firstly, certain roof shapes lack a unique and definitive definition. For instance, the saltbox shape presents difficulties in devising a suitable mathematical algorithm using only OSM information, necessitating additional estimation ideas or alternative data sources. Secondly, based on the results presented in Section 3, some roof shapes may not be reliably distinguishable in specific complex cases.

With respect to the last hypothesis, “whether the output models can archive considerable similarity to the real buildings”, this is achieved for simple buildings with complete information. However, other cases have numerous challenges, as highlighted in the preceding hypothesis points and sections. Challenges include uncontrolled building body parts, the appropriateness of the technique for non-rectangular footprints, potential inaccuracies in estimations for missing information based on studied tags, and the presence of tags that were not studied, among other factors.

## 5. Conclusions

As mentioned in Section 4, the technique applied to virtual rectangles is not universally suitable for non-rectangular footprints, indicating the need for further refinement and improvement in handling such cases. Additionally, another idea is put forth to augment the overall performance of this work.

For buildings where the virtual rectangle roof technique is not suitable, a complementary method is needed to address these challenges. In the future, a proposed enhancement of the methodology concerning buildings without a rectangular footprint will address the issue of indistinguishable roof shapes.

The technique improvement for non-rectangle footprints has a viable solution: creating new algorithms to replace complex orthogonal polygons with a combination of rectangles, as demonstrated in Figure 5 from the work of Sugihara et al. [34]. The key points in this solution are as follows:

- Use the minimum number of rectangles.
- Determine the rectangles with the maximum sum of areas, allowing for potential overlap between rectangles. The objective is to maximize the combined area of these rectangles without increasing the overall count of rectangles.
- Generate a 3D roof for each of these rectangles. Subsequently, these individual roofs are combined using the union operation to form the final composite roof.

This approach may be more suitable for buildings with diverse shapes of exterior borders, as demonstrated in existing applications [34]. However, to classify how the building roofs are constructed, whether through the subdivision of a rectangle (Section 2.3) or the union of multiple rectangles [34], additional information or estimation will be required.

In contrast, the introduced GLB 3D model transmission format has the potential to facilitate real-time modifications in the generated models. Subsequent efforts could explore its integration into the dynamic web interface of 3D modeling, transforming it from a static storage format to a platform capable of dynamic, real-time changes.

Additional future improvements could involve incorporating more sophisticated modeling techniques, such as machine learning algorithms, which could better adapt to diverse roof shapes and building complexities. Furthermore, integrating more granular geospatial data from sources like LiDAR or higher-resolution satellite imagery could enhance the algorithm’s ability to handle intricate architectural details.

In terms of practical applications, the proposed algorithm has significant potential in urban planning and smart city initiatives. It could be used to generate accurate 3D models for urban simulations, solar energy analysis, and urban heat island effect studies. The generated models can also be employed in augmented reality applications, providing a more immersive experience for urban visualization.

## Figures and Tables

**Table 1:** Parameters for shapes that do not need side length comparison.

Shape	Points 2D Coordinates	Roof Faces	Side Extension Faces
Pyramidal	$T=\frac{B_{0}+B_{2}}{2}$	$\left\{\begin{array}{c} \left({\dot{B}}_{0},{\dot{B}}_{1},\dot{T}\right)\\ \left({\dot{B}}_{1},{\dot{B}}_{2},\dot{T}\right)\\ \left({\dot{B}}_{2},{\dot{B}}_{3},\dot{T}\right)\\ \left({\dot{B}}_{3},{\dot{B}}_{0},\dot{T}\right) \end{array}\right.$	-
Skillion	$\begin{array}{c} T_{01}=B_{0}\\ T_{23}=B_{3} \end{array}$	$\left\{\begin{array}{c} \left({\dot{B}}_{1},{\dot{B}}_{2},{\dot{T}}_{23},{\dot{T}}_{01}\right) \end{array}\right.$	$\left\{\begin{array}{c} \left({\dot{B}}_{0},{\dot{B}}_{1},{\dot{T}}_{01}\right)\\ \left({\dot{B}}_{2},{\dot{B}}_{3},{\dot{T}}_{23}\right)\\ \left({\dot{B}}_{3},{\dot{B}}_{0},{\dot{T}}_{01},{\dot{T}}_{23}\right) \end{array}\right.$

**Table 2:** Parameters for shapes that do not need side length comparison.

Shape	Points 2D Coordinates	Roof Faces	Side Extension Faces
Hipped	$\begin{array}{cc} {\vec{v}}_{03}^{\prime }={\vec{v}}_{03}\cdot \frac{|{\vec{v}}_{01}|}{|{\vec{v}}_{03}|}\; & {\vec{v}}_{t}=\frac{{\vec{v}}_{01}+{\vec{v}}_{03}^{\prime }}{2}\\ T_{01}=B_{0}+{\vec{v}}_{t}\; & T_{23}=B_{2}-{\vec{v}}_{t} \end{array}$	$\left\{\begin{array}{c} \left({\dot{B}}_{0},{\dot{B}}_{1},{\dot{T}}_{01}\right)\\ \left({\dot{B}}_{1},{\dot{B}}_{2},{\dot{T}}_{23},{\dot{T}}_{01}\right)\\ \left({\dot{B}}_{2},{\dot{B}}_{3},{\dot{T}}_{23}\right)\\ \left({\dot{B}}_{3},{\dot{B}}_{0},{\dot{T}}_{01},{\dot{T}}_{23}\right) \end{array}\right.$	-
Half-hipped	$\begin{array}{cc} {\vec{v}}_{m}=\frac{HE}{2}\; & \;\cdot \;{\vec{v}}_{01}\\ M_{0}=B_{0}+{\vec{v}}_{m}\; & M_{1}=B_{1}-{\vec{v}}_{m}\\ M_{2}=B_{2}-{\vec{v}}_{m}\; & M_{3}=B_{3}+{\vec{v}}_{m}\\ {\vec{v}}_{03}^{\prime }={\vec{v}}_{03}\cdot \frac{|{\vec{v}}_{01}|}{|{\vec{v}}_{03}|}\;{\vec{v}}_{t}= & \;\frac{1-HE}{2}\cdot \left({\vec{v}}_{01}+{\vec{v}}_{03}^{\prime }\right)\\ T_{01}=M_{0}+{\vec{v}}_{t}\; & T_{23}=M_{2}-{\vec{v}}_{t} \end{array}$	$\left\{\begin{array}{c} \left({\dot{B}}_{1},{\dot{B}}_{2},{\dot{M}}_{2},{\dot{T}}_{23},{\dot{T}}_{01},{\dot{M}}_{1}\right)\\ \left({\dot{B}}_{3},{\dot{B}}_{0},{\dot{M}}_{0},{\dot{T}}_{01},{\dot{T}}_{23},{\dot{M}}_{3}\right)\\ \left({\dot{M}}_{0},{\dot{M}}_{1},{\dot{T}}_{01}\right)\\ \left({\dot{M}}_{2},{\dot{M}}_{3},{\dot{T}}_{23}\right) \end{array}\right.$	$\left\{\begin{array}{c} \left({\dot{B}}_{0},{\dot{B}}_{1},{\dot{M}}_{1},{\dot{M}}_{0}\right)\\ \left({\dot{B}}_{2},{\dot{B}}_{3},{\dot{M}}_{3},{\dot{M}}_{2}\right) \end{array}\right.$
Mansard	$\begin{array}{cc} {\vec{v}}_{01}^{\prime }=\frac{{\vec{v}}_{01}}{2}\;{\vec{v}}_{03}^{\prime } & \;={\vec{v}}_{03}\cdot \frac{|{\vec{v}}_{01}^{\prime }|}{|{\vec{v}}_{03}|}\\ {\vec{w}}_{0}={\vec{v}}_{03}^{\prime }+{\vec{v}}_{01}^{\prime }\; & {\vec{w}}_{1}={\vec{v}}_{03}^{\prime }-{\vec{v}}_{01}^{\prime }\\ T_{01}=B_{0}+{\vec{w}}_{0}\; & T_{23}=B_{2}-{\vec{w}}_{0}\\ M_{0}=T_{01}-MP\cdot {\vec{w}}_{0}\; & M_{1}=T_{01}-MP\cdot {\vec{w}}_{1}\\ M_{2}=T_{23}+MP\cdot {\vec{w}}_{0}\; & M_{3}=T_{23}+MP\cdot {\vec{w}}_{1} \end{array}$	$\left\{\begin{array}{c} \left({\dot{B}}_{0},{\dot{B}}_{1},{\dot{M}}_{1},{\dot{M}}_{0}\right)\\ \left({\dot{B}}_{1},{\dot{B}}_{2},{\dot{M}}_{2},{\dot{M}}_{1}\right)\\ \left({\dot{B}}_{2},{\dot{B}}_{3},{\dot{M}}_{3},{\dot{M}}_{2}\right)\\ \left({\dot{B}}_{3},{\dot{B}}_{0},{\dot{M}}_{0},{\dot{M}}_{3}\right)\\ \left({\dot{M}}_{0},{\dot{M}}_{1},{\dot{T}}_{01}\right)\\ \left({\dot{M}}_{1},{\dot{M}}_{2},{\dot{T}}_{23},{\dot{T}}_{01}\right)\\ \left({\dot{M}}_{2},{\dot{M}}_{3},{\dot{T}}_{23}\right)\\ \left({\dot{M}}_{3},{\dot{M}}_{0},{\dot{T}}_{01},{\dot{T}}_{23}\right) \end{array}\right.$	-

**Table 3:** Parameters for shapes that depend on an OSM parameter.

Shape	Points 2D Coordinates	Roof Faces	Side Extension Faces
Gabled	$\begin{array}{c} T_{01}=\frac{B_{0}+B_{1}}{2}\;T_{23}=\frac{B_{2}+B_{3}}{2} \end{array}$	$\left\{\begin{array}{c} \left({\dot{B}}_{1},{\dot{B}}_{2},{\dot{T}}_{23},{\dot{T}}_{01}\right)\\ \left({\dot{B}}_{3},{\dot{B}}_{0},{\dot{T}}_{01},{\dot{T}}_{23}\right) \end{array}\right.$	$\left\{\begin{array}{c} \left({\dot{B}}_{0},{\dot{B}}_{1},{\dot{T}}_{01}\right)\\ \left({\dot{B}}_{2},{\dot{B}}_{3},{\dot{T}}_{23}\right) \end{array}\right.$
Gambrel	$\begin{array}{cc} {\vec{v}}_{01}^{\prime }=\frac{1-GP}{2}\; & \cdot \;{\vec{v}}_{01}\\ M_{0}=B_{0}+{\vec{v}}_{01}^{\prime }\; & M_{1}=B_{1}-{\vec{v}}_{01}^{\prime }\\ M_{2}=B_{2}-{\vec{v}}_{01}^{\prime }\; & M_{3}=B_{3}+{\vec{v}}_{01}^{\prime }\\ T_{01}=\frac{B_{0}+B_{1}}{2}\; & T_{23}=\frac{B_{2}+B_{3}}{2}\\ \; \end{array}$	$\left\{\begin{array}{c} \left({\dot{B}}_{1},{\dot{B}}_{2},{\dot{M}}_{2},{\dot{M}}_{1}\right)\\ \left({\dot{B}}_{3},{\dot{B}}_{0},{\dot{M}}_{0},{\dot{M}}_{3}\right)\\ \left({\dot{M}}_{1},{\dot{M}}_{2},{\dot{T}}_{23},{\dot{T}}_{01}\right)\\ \left({\dot{M}}_{3},{\dot{M}}_{0},{\dot{T}}_{01},{\dot{T}}_{23}\right) \end{array}\right.$	$\left\{\begin{array}{c} \left({\dot{B}}_{0},{\dot{B}}_{1},{\dot{M}}_{1},{\dot{T}}_{01},{\dot{M}}_{0}\right)\\ \left({\dot{B}}_{2},{\dot{B}}_{3},{\dot{M}}_{3},{\dot{T}}_{23},{\dot{M}}_{2}\right) \end{array}\right.$

**Table 4:** Parameters for shapes using a single alternative axis.

Shape	Cross Values	Descending Level Function
Skillion	$x_{C}^{\prime }=⌀$	$DLF\left(x^{\prime }\right)=\frac{1-x^{\prime }}{2}$
Gabled	$x_{C}^{\prime }=\left\{0\right\}$	$DLF\left(x^{\prime }\right)=\left|x^{\prime }\right|$
Gambrel	$x_{C}^{\prime }=\left\{-GP,0,GP\right\}$	$\;DLF\left(x^{\prime }\right)=\;\left\{\begin{array}{cc} \frac{1-GP}{GP}\cdot \left|x^{\prime }\right|, & |x^{\prime }|\leq GP\\ 1-\frac{GP}{1-GP}\cdot (1-|x^{\prime }\left|\right), & |x^{\prime }|>GP \end{array}\right.$

**Table 5:** Special alternative values for shapes using the double alternative axis.

Shape	Special Value 1	Special Value 2
Hipped	$x_{H}^{\prime }=1-\frac{reduction_{y^{\prime }}}{reduction_{x^{\prime }}}$	
Half-hipped	$y_{H}^{\prime }=1-HE$	$x_{H}^{\prime }=1-y_{H}^{\prime }\cdot \frac{reduction_{y^{\prime }}}{reduction_{x^{\prime }}}$
Mansard	$x_{H}^{\prime }=1-\frac{reduction_{y^{\prime }}}{reduction_{x^{\prime }}}$	$x_{M}^{\prime }=x_{H}^{\prime }+MP\cdot \frac{reduction_{y^{\prime }}}{reduction_{x^{\prime }}}$

**Table 6:** Virtual geometries for shapes using the double alternative axis.

Shape	Cross Segments	Alternative Face Positions
Pyramidal	$\left\{\begin{array}{c} \bar{\left(-1,-1);(1,1\right)}\\ \bar{\left(-1,1);(1,-1\right)} \end{array}\right.$	$\left\{\begin{array}{c} \left(-1,-1);(-1,1);(0,0\right)\\ \left(-1,1);(1,1);(0,0\right)\\ \left(1,1);(1,-1);(0,0\right)\\ \left(1,-1);(-1,-1);(0,0\right) \end{array}\right.$
Hipped	$\left\{\begin{array}{c} \bar{\left(-1,-1\right);\left(-x_{H}^{\prime },0\right)}\\ \bar{\left(-1,1\right);\left(-x_{H}^{\prime },0\right)}\\ \bar{\left(-x_{H}^{\prime },0\right);\left(x_{H}^{\prime },0\right)}\\ \bar{\left(x_{H}^{\prime },0\right);\left(1,-1\right)}\\ \bar{\left(x_{H}^{\prime },0\right);\left(1,1\right)} \end{array}\right.$	$\left\{\begin{array}{c} \left(-1,-1\right);\left(-1,1\right);\left(-x_{H}^{\prime },0\right)\\ \left(1,1\right);\left(1,-1\right);\left(x_{H}^{\prime },0\right)\\ \left(1,-1\right);\left(-1,-1\right);\left(-x_{H}^{\prime },0\right);\left(x_{H}^{\prime },0\right)\\ \left(-1,1\right);\left(1,1\right);\left(x_{H}^{\prime },0\right);\left(-x_{H}^{\prime },0\right) \end{array}\right.$
Half-hipped	$\left\{\begin{array}{c} \bar{\left(-1,-y_{H}^{\prime }\right);\left(-x_{H}^{\prime },0\right)}\\ \bar{\left(-1,y_{H}^{\prime }\right);\left(-x_{H}^{\prime },0\right)}\\ \bar{\left(-x_{H}^{\prime },0\right);\left(x_{H}^{\prime },0\right)}\\ \bar{\left(x_{H}^{\prime },0\right);\left(1,-y_{H}^{\prime }\right)}\\ \bar{\left(x_{H}^{\prime },0\right);\left(1,y_{H}^{\prime }\right)} \end{array}\right.$	$\left\{\begin{array}{c} \left(-1,-y_{H}^{\prime }\right);\left(-1,y_{H}^{\prime }\right);\left(-x_{H}^{\prime },0\right)\\ \left(1,y_{H}^{\prime }\right);\left(1,-y_{H}^{\prime }\right);\left(x_{H}^{\prime },0\right)\\ \left(1,-1\right);\left(-1,-1\right);\left(-1,-y_{H}^{\prime }\right);\left(-x_{H}^{\prime },0\right);\left(x_{H}^{\prime },0\right);\left(1,-y_{H}^{\prime }\right)\\ \left(-1,1\right);\left(1,1\right);\left(1,y_{H}^{\prime }\right);\left(x_{H}^{\prime },0\right);\left(-x_{H}^{\prime },0\right);\left(-1,y_{H}^{\prime }\right) \end{array}\right.$
Mansard	$\left\{\begin{array}{c} \bar{\left(-1,-1\right);\left(-x_{H}^{\prime },0\right)}\\ \bar{\left(-1,1\right);\left(-x_{H}^{\prime },0\right)}\\ \bar{\left(-x_{H}^{\prime },0\right);\left(x_{H}^{\prime },0\right)}\\ \bar{\left(x_{H}^{\prime },0\right);\left(1,-1\right)}\\ \bar{\left(x_{H}^{\prime },0\right);\left(1,1\right)}\\ \bar{\left(-x_{M}^{\prime },-MP\right);\left(x_{M}^{\prime },-MP\right)}\\ \bar{\left(x_{M}^{\prime },-MP\right);\left(x_{M}^{\prime },MP\right)}\\ \bar{\left(x_{M}^{\prime },MP\right);\left(-x_{M}^{\prime },MP\right)}\\ \bar{\left(-x_{M}^{\prime },MP\right);\left(-x_{M}^{\prime },-MP\right)} \end{array}\right.$	$\left\{\begin{array}{c} \left(-1,-1\right);\left(-1,1\right);\left(-x_{M}^{\prime },MP\right);\left(-x_{M}^{\prime },-MP\right)\\ \left(-x_{M}^{\prime },-MP\right);\left(-x_{M}^{\prime },MP\right);\left(-x_{H}^{\prime },0\right)\\ \left(1,1\right);\left(1,-1\right);\left(x_{M}^{\prime },-MP\right);\left(x_{M}^{\prime },MP\right)\\ \left(x_{M}^{\prime },MP\right);\left(x_{M}^{\prime },-MP\right);\left(x_{H}^{\prime },0\right)\\ \left(1,-1\right);\left(-1,-1\right);\left(-x_{M}^{\prime },-MP\right);\left(x_{M}^{\prime },-MP\right)\\ \left(x_{M}^{\prime },-MP\right);\left(-x_{M}^{\prime },-MP\right);\left(-x_{H}^{\prime },0\right);\left(x_{H}^{\prime },0\right)\\ \left(-1,1\right);\left(1,1\right);\left(x_{M}^{\prime },MP\right);\left(-x_{M}^{\prime },MP\right)\\ \left(-x_{M}^{\prime },MP\right);\left(x_{M}^{\prime },MP\right);\left(x_{H}^{\prime },0\right);\left(-x_{H}^{\prime },0\right) \end{array}\right.$

**Table 7:** Descending level functions for shapes using the double alternative axis.

Shape	${\mathrm{DLF}}_{x}\left(x^{\prime }\right)=\cdots$	${\mathrm{DLF}}_{y}\left(y^{\prime }\right)=\cdots$
Hipped	$\left\{\begin{array}{cc} 0, & |x^{\prime }|\leq x_{H}^{\prime }\\ \frac{|x^{\prime }|-x_{H}^{\prime }}{1-x_{H}^{\prime }}, & |x^{\prime }|>x_{H}^{\prime } \end{array}\right.$	$|y^{\prime }|$
Pyramidal	$|x^{\prime }|$	$|y^{\prime }|$
Half-hipped	$\left\{\begin{array}{cc} 0, & |x^{\prime }|\leq x_{H}^{\prime }\\ \left(\right|x^{\prime }\left|-x_{H}^{\prime }\right)\cdot \frac{reduction_{x^{\prime }}}{reduction_{y^{\prime }}}, & |x^{\prime }|>x_{H}^{\prime } \end{array}\right.$	$|y^{\prime }|$
Mansard	$\left\{\begin{array}{cc} 0, & |x^{\prime }|\leq x_{H}^{\prime }\\ DLF_{y}\left(\frac{|x^{\prime }|-x_{H}^{\prime }}{1-x_{H}^{\prime }}\right), & |x^{\prime }|>x_{H}^{\prime } \end{array}\right.$	$\left\{\begin{array}{cc} \frac{1-MP}{MP}\cdot \left|y^{\prime }\right|, & |y^{\prime }|\leq MP\\ 1-\frac{MP}{1-MP}\cdot (1-|y^{\prime }\left|\right), & |y^{\prime }|>MP \end{array}\right.$

## Data Availability

Data are contained within the article.

## Abbreviations

The following abbreviations are used in this manuscript:

OSM	OpenStreetMap
WGS	World Geodetic System
LoD	Level of Detail
LiDAR	Light Detection and Ranging
DLG	Digital Line Graphic
HE	Hip Elevation
MP	Mansard Portion
GP	Gambrel Portion
DLF	Descending level function
GLB	Graphics Library Binary
